# Effect of Aerobic Exercise on Improving Symptoms of Individuals With Schizophrenia: A Single Blinded Randomized Control Study

**DOI:** 10.3389/fpsyt.2018.00167

**Published:** 2018-05-15

**Authors:** Peng-Wei Wang, Huang-Chi Lin, Chwen-Yng Su, Ming-De Chen, Kuo C. Lin, Chih-Hung Ko, Cheng-Fang Yen

**Affiliations:** ^1^Psychiatry, Kaohsiung Medical University Hospital, Kaohsiung, Taiwan; ^2^Department of Psychiatry, Faculty of Medicine and Graduate Institute of Medicine, College of Medicine, Kaohsiung Medical University, Kaohsiung, Taiwan; ^3^Department of Occupational Therapy, College of Health Sciences, Kaohsiung Medical University, Kaohsiung, Taiwan; ^4^Division of Physical and Health Education, Center for General Education, National Sun Yat-sen University, Kaohsiung, Taiwan

**Keywords:** schizophrenia, psychosis, aerobic exercise, negative symptoms, positive symptoms

## Abstract

**Introduction:** Antipsychotic treatment can improve the symptoms of schizophrenia; however, residual symptoms after antipsychotic treatment are frequent. The effects of exercise on the symptoms of schizophrenic patients under antipsychotic treatment are inconclusive. The aim of this randomized case-control study was to examine the effects of aerobic exercise (AE) on the symptoms of schizophrenic patients receiving antipsychotic treatment.

**Methods:** In total, 33 and 29 participants being treated with antipsychotics for schizophrenia were randomly assigned into the aerobic exercise (AE) group and the control group, respectively. The severities of schizophrenic symptoms were measured using the Chinese version of the Positive and Negative Syndrome Scale (PANSS) before, immediately after, and 3 months after the intervention in both groups.

**Results:** In total, 24 participants (72.7%) in the AE group and 22 (75.9%) in the control group completed the study. The results indicated that the severities of positive symptoms and general psychopathology in the AE group significantly decreased during the 12 weeks of intervention but did not further significantly change during the 3-month follow-up period. The severities of negative symptoms in the AE group decreased significantly after 12 weeks of intervention and continued decreasing during the 3-month follow-up period. Interaction effects between time and group on the severities of symptoms on the negative and general psychopathology scales were observed.

**Conclusion:** AE can improve the severities of symptoms on the negative and general psychopathology scales in individuals with schizophrenia being treated with antipsychotics.

## Introduction

Schizophrenia affects approximately 1% of the general population and is a major mental health issue worldwide (1). The clinical presentation of schizophrenia is associated with positive and negative symptoms. Studies of prodromal symptoms of schizophrenia revealed that negative symptoms are evident at the time of the first psychotic episode (2, 3). Furthermore, more than half of individuals with schizophrenia have one or more negative symptoms (4), and 25–30% of individuals with schizophrenia have enduring negative symptoms (5, 6). The severities of negative symptoms may be associated with psychosocial impairment and work performance (7). In addition, negative symptoms contribute greatly to disability in patients with schizophrenia (8). A previous study showed that negative symptoms play a role in recovery in patients with schizophrenia (9). Negative symptoms are also the major source of caregiver burden for patients' families (10).

Antipsychotics are the cornerstone of treatment of schizophrenia. However, a study of the effectiveness of antipsychotics for the treatment of schizophrenia and schizophreniform disorder reported symptom reductions of only around 60% after antipsychotic treatment (11). Furthermore, only 23% of antipsychotic-treated schizophrenia patients achieve a good response (12). These results implied that while antipsychotics are essential in treating schizophrenia, interventions other than antipsychotic treatment, such as exercise, may be needed to achieve better outcomes. In addition, unlike positive symptoms, which are amenable to antipsychotic drug treatment (13), antipsychotic drugs and other psychotropic drugs have been demonstrated to have limited effects in terms of reducing negative symptoms (12, 14). This highlights the importance of exploring whether non-pharmacological interventions can reduce the levels of negative symptoms.

The benefits of exercise are evident in people with illnesses, and research has shown that exercise contributes to the treatment of several chronic illnesses (15). In terms of the biological mechanism, exercise can increase the levels of brain-derived neurotropic factor (BDNF) and other neurotrophic factors, and correct dysfunction of the hypothalamic–pituitary–adrenal system (16, 17). Reviews of the effects of exercise in patients with anxiety disorder and depressive disorder indicated that in comparison with drug treatment, exercise monotherapy exhibited a lesser effect, but a combination of exercise and pharmacotherapy resulted in greater improvement than drug treatment alone (18, 19).

In patients with schizophrenia, exercise has been shown to improve cognitive skills and physical health (20, 21). Vogel et al. reviewed studies of the effects of exercise on negative symptoms in schizophrenic patients, and concluded that an effect could not be confirmed owing to the poor quality of the included studies (22). Methodological limitations lead to difficulty in drawing firm conclusions from exercise intervention studies in patients with mental illnesses (23), common limitations being the lack of a blind assessment of outcome, low adherence to the exercise program, lack of follow-up assessment, and randomization not being undertaken (23). Furthermore, Pearsall et al. (24) reviewed studies of the effect of exercise in schizophrenia, and argued that limited improvement only may result from exercise of an insufficient intensity. To date, evidence showing that regular high-intensity aerobic exercise (AE) decreases the intensity of key symptoms of schizophrenia is scarce. Therefore, the aim of this randomized case-control study was to examine the blind-assessed augmentative effect of high-intensity AE on the symptoms of schizophrenic patients receiving antipsychotic treatment.

## Methods

### Study design

This study, which was conducted between 2012 and 2015, was a single-blind randomized case-control trial. After screening and explaining the process of this study, all participants signed an informed consent form, following which they were randomly allocated into an aerobic exercise (AE) group or a stretching control group. Patients in both the AE and stretching groups underwent three evaluations in total, at baseline, at the end of the intervention, and at the 3-month follow-up point. The psychiatrist who evaluated the participants was blind to their group assignments. The protocol was approved by the Institutional Review Board of Kaohsiung Medical University.

The inclusion criteria were as follows: (1) participants who fulfilled the DSM-IV-TR criteria for schizophrenia, assessed using the Structured Clinical Interview for DSM-IV Axis-I Disorders (25), and who took antipsychotic drugs without changing the medication or dosage for more than 3 months prior to entry into this study; and (2) participants who were assessed using the modified Bruce protocol (26) and deemed physically well enough to exercise. The exclusion criteria included (1) a duration of schizophrenia less than 1 year before entry into the study; (2) participants with any neurological illness or psychiatric condition other than schizophrenia; (3) participants who were pregnant or breast feeding; and (4) participants with current or previous substance use.

### Aerobic exercise intervention and stretching-toning control program

The intensity of AE intervention was based on each individual's age-adjusted maximum heart rate (maximum heart rate: 220-age). Each AE session included 5 min of walking for a warm-up, followed by 30 min of AE, then finally a 5-min cool-down period, i.e., 40 min in total. To ensure that participants were exercising safely at their target intensity, Polar HR monitors (Polar RS 300HR, Polar Electro Oy, Finland) were used to monitor exercise intensity throughout the 12-week training program under one-to-one supervision. The stretching and toning control program consisted of a 30-min recorded program of 14 exercise routines, including a 3-min warm-up, 25-min flexibility, toning and balance exercises designed to use all major muscle groups of the upper and lower extremities, and a 2-min cool-down exercise performed to music. The supervisor gave every participant one-to-one monitoring through every session to maintain fidelity. The attendance goal for both groups was five times per week. Participants of both groups were scheduled to contact the trainer three times per week for 3 months to ensure that their rate of attendance exceeded 60%. Thus, all participants completed at least 36 sessions.

### Outcome measure

#### Mandarin chinese version of the positive and negative syndrome scale (MC-PANSS)

The 30-item PANSS is designed to evaluate the severities of positive symptoms, negative symptoms and general psychopathology in patients with schizophrenia (27). The symptom severity for each item is rated according to which anchoring point in the 7-point scale (1 = absent; 7 = extreme) best describes the presentation of the symptom. Higher scores indicate that patients are experiencing more severe symptoms. The MC-PANSS has been demonstrated to be of good reliability and validity (28). The MC-PANSS was used in this study to measure the severity of schizophrenia at baseline, at the end of the intervention, and at the 3-month follow-up point.

### Statistical analyses

Data analysis was performed using SPSS 23.0 Software (29). Baseline characteristics and symptom severities at each follow-up point were compared between the two groups using the χ^2^ test and Student's *t*-test. Intent-to-treat analysis was applied to determine differences in symptom severities between the two groups using a generalized estimating equation (GEE) (30). The correlation model was auto-regressive. Inferences were made at the 0.05 level of significance for inferential statistical procedures. The Hochberg sequential procedure was used for multiple comparison correction (31).

## Results

**Figure 1:**
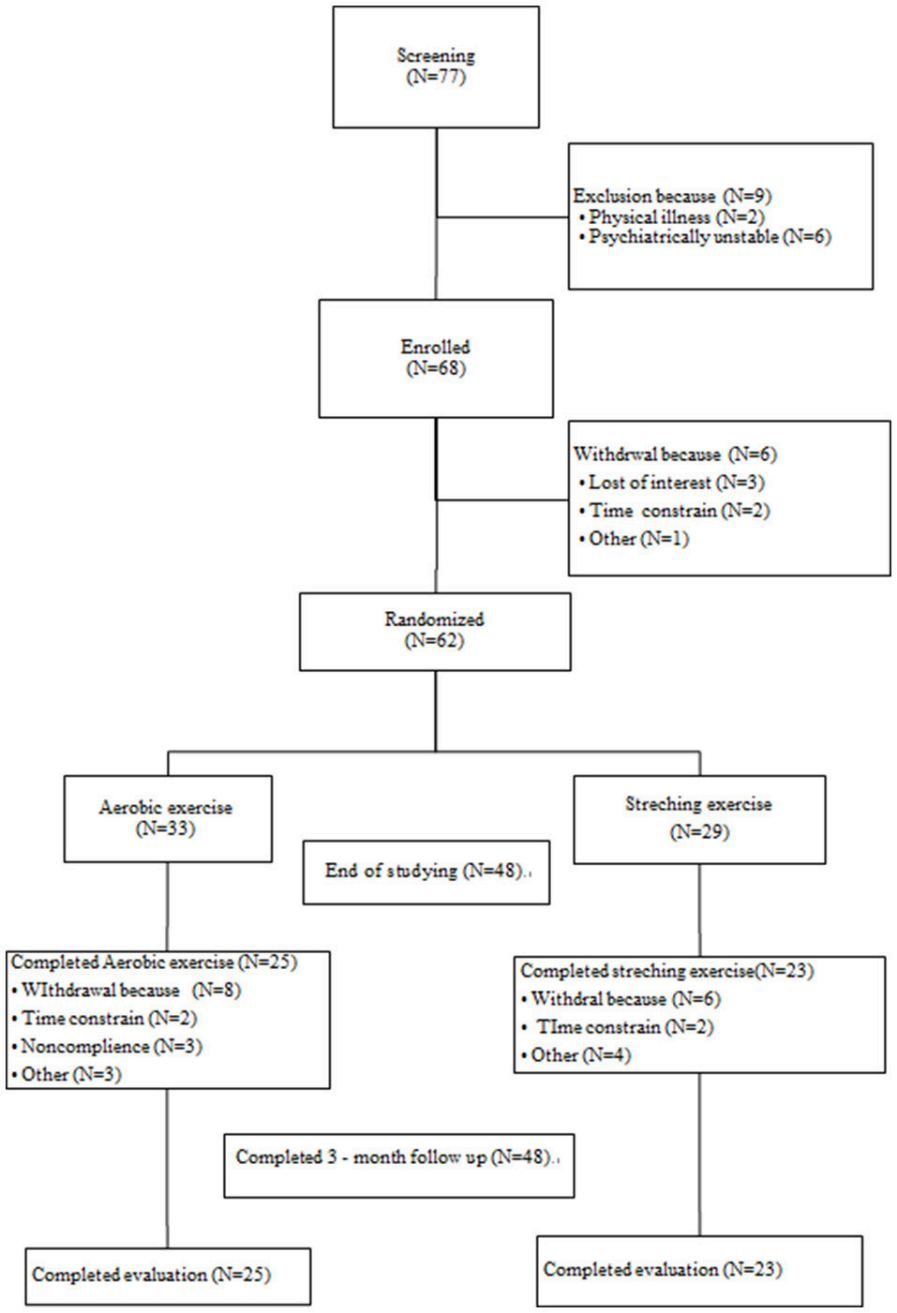
CONSOR diagram.

In total, 77 participants were screened; nine did not fulfill the inclusion criteria, and six were withdrawn prior to randomization. A total of 62 participants were therefore randomly assigned into either the aerobic exercise (AE) group (33 participants) or the control group (29 participants). Nine (27.27%) and seven participants (24.14%) in the AE and control groups did not complete the study, respectively. Figure 1 shows the study flowchart and the reasons for drop-out. No participant dropped out during the follow-up period. There were no differences in the baseline characteristics between the completers and non-completers.

There were 18 female patients (55.55%) in the AE group and 14 (48.28%) in the control group. The gender difference between the two groups was not significant (χ^2^ = 0.243; *p* = 0.799). The mean age of the AE group and control group was 38.3 years (standard deviation [SD]: 8.34 years) and 38.72 years (SD: 8.62 years), respectively. The age difference was also not significant between the two groups (*t* = −0.195; *p* = 0.846). There was no difference in the age of onset of schizophrenia between the AE group and control group (23.91 ± 7.24 years vs. 23.86 ± 7.45 years, *t* = 0.029, *p* = 0.994).The mean dosage (chlorpromazine equivalent) of antipsychotics in the AE group and control group did not differ at baseline (540.65 ± 485.65 mg/day vs. 420.34 ± 314.45 mg/day, *t* = 1.171, *p* = 0.878), at the end of the intervention (532.43 ± 479.81 mg/day vs. 423.72 ± 324.57 mg/day, *t* = 0.926, *p* = 0.819), or after the 3-month follow-up period (532.43 ± 479.81 mg/day vs. 423.72 ± 324.57 mg/day, *t* = 0.926, *p* = 0.819). The average overall duration of training was 1408.50 min (SD: 246 min). The mean percentage of patients who met the prescribed level of intensity of training was 100%.

Comparisons of the severities of positive and negative symptoms and general psychopathology at baseline, at the end of the intervention, and 3 months later between the AE group and control group are presented in Table 1. In the AE group, the severities of negative symptoms were significantly lower than those of the control group at the end of the intervention and at the 3-month follow-up point.

**Table 1:** Severities of symptoms at baseline, the end of the intervention, and the 3-month follow-up point in the aerobic exercise (AE) and control groups.

	**Baseline**			**End of intervention**			**3-month follow-up**		
	**AE group Mean (SD)**	**Control group Mean (SD)**	***p***	**AE group Mean (SD)**	**Control group Mean (SD)**	***p***	**AE group Mean (SD)**	**Control group Mean (SD)**	***p***
Positive symptoms	18.06 (6.44)	19.21 (7.43)	0.518	14.12 (5.03)	16.00 (6.30)	0.257	14.38 (4.57)	15.36 (6.57)	0.554
Negative symptoms	21.88 (8.77)	21.07 (7.90)	0.705	17.28 (7.19)	21.87 (8.19)	0.044	15.33 (5.61)	22.55 (9.87)	0.004
General psychopathology	41.09 (17.43)	37.17 (11.15)	0.303	29.87 (9.96)	33.60 (11.85)	0.248	29.82 (8.19)	34.36 (15.76)	0.157
Total PANSS score	81.03 (24.96)	77.44 (20.29)	0.541	62.62 (20.07)	71.47 (20.14)	0.138	58.50 (16.87)	71.26 (30.17)	0.097

*PANSS: Positive and Negative Syndrome Scale*.

**Figure 2:**
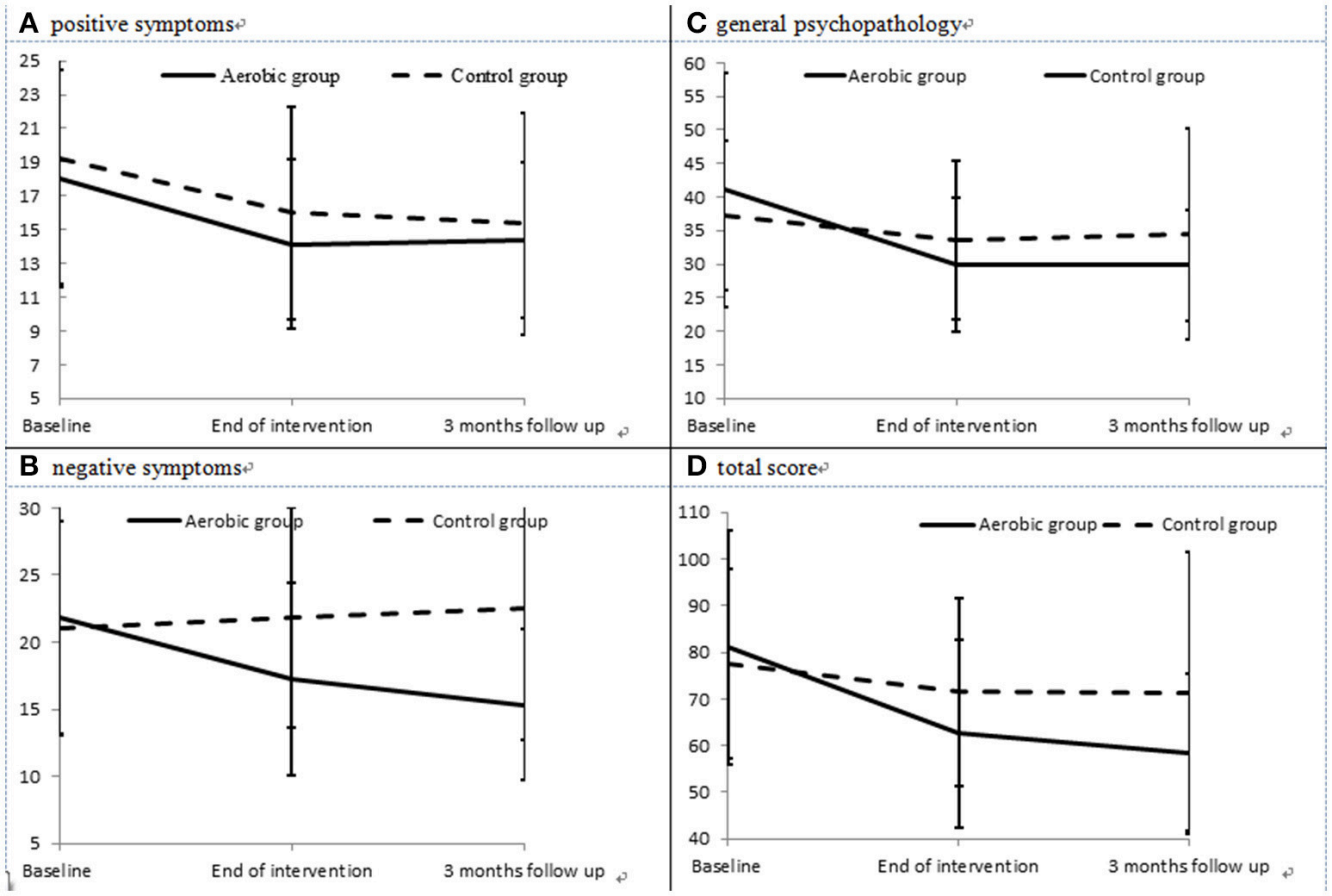
Severities of positive **(A)** and negative symptoms **(B)**, general psychopathology **(C)**, and total PANSS score **(D)** in the aerobic exercise (AE) group and control group at each evaluation point.

The severities of positive symptoms (Figure 2A) in the AE group decreased significantly between baseline and the end of the intervention (paired *t* = 3.06, *p* = 0.005), but did not differ between the end of the intervention and the 3-month follow-up point (paired *t* = −0.17, *p* = 0.864). The severities of negative symptoms (Figure 2B) in the AE group decreased significantly between baseline and the end of the intervention (paired *t* = 2.75, *p* = 0.012), and also decreased between the end of the intervention and the 3-month follow-up point (paired *t* = 2.14, *p* = 0.043). In addition, the AE group exhibited an improved severity of general psychopathology (Figure 2C) from baseline to the end of the intervention (paired *t* = 3.98, *p* = 0.001), but no further improvement was observed during the 3-month follow-up period (paired *t* = 1.00, *p* = 0.332). Meanwhile, the control group did not exhibit any significant improvements in positive or negative symptoms, nor in general psychopathology, during the intervention or the follow-up period. The effect size (Cohen's d) for the changes in positive symptoms, negative symptoms, and general psychopathology between baseline and the end of the intervention was −0.09, −0.97, and −0.64, respectively.

The results of intent-to-treat analysis of the differences in the effects of intervention on symptoms between the AE group and the control group are presented in Table 2. The AE group showed better improvement in negative symptoms, general psychopathology and the total PANSS score (Figure 2D) than the control group, and interaction effects between time and group on the severity of negative symptoms, general psychopathology and the total PANSS score were observed after controlling for the effects of gender and age.

**Table 2:** Differences in the effects of treatment on symptoms between the aerobic exercise (AE) group and control group examined using intent-to-treat analysis.

	**Positive symptoms**		**Negative symptoms**		**General psychopathology**		**Total**	
	**Coefficient**	***p***	**Coefficient**	***p***	**Coefficient**	***p***	**Coefficient**	***p***
Gender^a^	0.23	0.861	0.92	0.565	−4.19	0.123	−3.83	0.428
Age	0.09	0.224	−0.11	0.315	−0.25	0.085	−0.30	0.226
Group	−1.27	0.592	4.99	0.106	6.96	0.135	11.42	0.155
Time	−1.91	0.009	0.92	0.462	−1.43	0.292	−3.16	0.365
Group*Time	0.04	0.965	−4.39	0.003	−3.64	0.050	−8.66	0.029

*PANSS: Positive and Negative Syndrome Scale*.

a *Female as reference*.

The results of examining the ability of symptoms at intake to predict the effect of treatment in the AE group using intent-to-treat analysis are presented in Table 3, and showed that participants with more severe symptoms at intake exhibited greater improvement after the intervention, as the interactions between time and baseline symptoms were negative.

**Table 3:** Ability of symptoms at intake to predict treatment effect in the aerobic exercise (AE) group.

	**Positive symptoms**		**Negative symptoms**		**General psychopathology**		**Total**	
	**Coefficient**	***p***	**Coefficient**	***p***	**Coefficient**	***p***	**Coefficient**	***p***
Gender (female as reference)	−1.34	0.047	0.35	0.767	−1.13	0.473	−2.17	0.385
Age	0.05	0.215	−0.01	0.913	0.06	0.495	0.14	0.385
Time	3.42	0.001	6.84	< 0.001	10.38	< .001	18.31	< 0.001
Positive symptoms at intake	1.19	< 0.001						
Negative symptoms at intake			1.32	< 0.001				
General psychopathology at intake					1.31	< 0.001		
Total PANSS score at intake							1.27	< 0.001
Positive symptoms at intake*Time	−0.25	< 0.001						
Negative symptoms at intake*Time			−0.36	< 0.001				
General psychopathology at intake*Time					−0.35	< 0.001		
Total PANSS score at intake*Time							−0.30	< 0.001

*PANSS: Positive and Negative Syndrome Scale*.

## Discussion

There were several interesting findings of this study. First, AE was found to improve negative symptoms, general psychopathology and the total PANSS score in patients with schizophrenia. Second, the improvement in negative symptoms continued after cessation of the AE intervention. Third, the baseline severity of negative symptoms, general psychopathology and total PANSS score were found to predict improvement during AE intervention.

Schizophrenia is a chronic illness that places a high burden on society and families, as patients usually have psychosocial dysfunction (32). Drug treatment can improve the outcome for schizophrenia patients, but suboptimal outcomes are still very frequent in schizophrenia patients undergoing treatment with antipsychotics (33). The results of this study showed that AE can improve negative symptoms and general psychopathology during the period of intervention and further improve negative symptoms during the 3-month follow-up period in schizophrenic patients receiving antipsychotic treatment. One of the possible ways in which AE might improve schizophrenic symptoms is that exercise may increase the drug level by changing the pharmacokinetics of antipsychotics, e.g., changing the drug distribution or reducing drug elimination (34).

Research has shown that moderate exercise for 60 min a day, three times a week, can promote health and improve hypertension (35). Regarding mental illnesses, exercise has been shown to benefit patients with major depressive disorder, bipolar disorder and anxiety disorder (19, 36, 37). Therefore, AE itself may improve schizophrenic symptoms, and not just alter the pharmacokinetics of drugs used in treatment. A previous study found that AE could increase the level of brain-derived neurotropic factor (BDNF) in patients with schizophrenia (38). Meanwhile, another previous study showed that AE can increase the hippocampus volume in patients with schizophrenia (39). Furthermore, the serum BDNF level is negatively associated with symptoms of drug-naïve schizophrenia (40), and a low serum BDNF level may contribute to the psychopathology of schizophrenia (41). These results supported the idea that AE may directly improve the symptoms of schizophrenia by enhancing neurotrophic and neuroprotective mechanisms.

Moreover, the improvements in mental illnesses brought about by AE have been proposed to be related to an exercise-induced influence on the hypothalamic-pituitary-adrenal axis (HPA) (42). HPA dysfunction is an important characteristic in schizophrenia (43), and patients with schizophrenia may therefore benefit from exercise because it can help to regulate the HPA (16). This may be another reason for which patients with schizophrenia see improvement after intervention, and that improvement still persists at the 3-month follow-up point.

A previous study found that the use of antipsychotics for the treatment of negative symptoms had only a minimal treatment effect, and was not as effective as their use for the treatment of positive symptoms (44). Our results showed that AE can improve negative symptoms in patients with schizophrenia. The current hypothesis regarding treatment for negative symptoms focuses on dysfunction of glutamate (45): enhancing brain glutamate function has been demonstrated to have the potential to reduce negative symptoms in patients with schizophrenia (46). In an animal model, exercise was demonstrated to increase the use of brain glutamate (47), and in humans, the level of brain glutamate can increase after vigorous exercise (48). Therefore, increasing glutamate in the brain may be the mechanism by which AE can reduce negative symptoms.

In this study, we also found that the more severe the symptoms of schizophrenia at intake, the greater the improvement that patients can achieve from AE. This result demonstrated that a more severe psychopathology is an indicator of greater improvement during AE intervention in patients with schizophrenia. There were several limitations of this study. First, the small sample size may limit the generalizability of our findings. Second, we did not ascertain whether the participants continued AE or not during the period of follow-up. Third, the role of fitness in the effects of AE on symptoms of schizophrenia warrants further examination.

## Conclusion

The results of this study indicate that AE can improve negative symptoms and general psychopathology in antipsychotic-treated patients with schizophrenia, and this improvement in symptoms can be maintained. Regarding negative symptoms, improvement persists beyond the period of AE intervention. In addition, patients with more severe symptoms at baseline may attain greater improvement via AE. These results imply that AE could be a good non-pharmacotheraputic intervention for antipsychotic-treated patients with schizophrenia.

## Author contributions

All authors listed have made a substantial, direct and intellectual contribution to the work, and approved it for publication.

### Conflict of interest statement

The authors declare that the research was conducted in the absence of any commercial or financial relationships that could be construed as a potential conflict of interest.
